# Clinical Experience Using Vitamin D and Analogs in the Treatment of Myelodysplasia and Acute Myeloid Leukemia: A Review of the Literature

**DOI:** 10.1155/2012/125814

**Published:** 2012-07-30

**Authors:** Jonathan S. Harrison, Alexander Bershadskiy

**Affiliations:** ^1^Division of Hematology, Department of Medicine, Robert Wood Johnson Medical School, New Brunswick, NJ 08901, USA; ^2^The Cancer Institute of New Jersey, University of Medicine and Dentistry of New Jersey, New Brunswick, NJ 08901, USA

## Abstract

Despite progress in understanding the biology of acute myeloid leukemia (AML), and despite advances in treatment, the majority of patients with AML die from the disease. The observation that Vitamin D can induce AML blast cells *in vitro* to differentiate along the monocytic lineage was made 30 years ago; however, it remains to translate this into a clinically meaningful strategy. This is a review of published clinical experience regarding the use of Vitamin D and its analogs, either alone or in combination with other agents, to treat AML. In many of these reports, investigators included patients with myelodysplasia (MDS) as well as AML patients in their treatment cohorts; therefore reports of Vitamin D and its analogs in treating MDS are included. This review documents heterogeneity in selection criteria for patients treated in these studies, the spectrum of Vitamin D analogs used in various studies, and the differing dosing strategies employed by investigators. Despite examples of occasional clinical efficacy, barriers remain to the successful application of Vitamin D in the treatment of MDS and AML. These include the lack of definition of a particularly sensitive target population, and the as yet unknown optimal choice of Vitamin D analog and dosing schedule.

## 1. Introduction

Despite significant progress over the past several decades in understanding the biology of acute myeloid leukemia (AML) and despite important advances in treatment approaches, the majority of patients who develop acute myeloid leukemia will still die from their disease. A recent analysis by Southwest Oncology Group investigators of data from 1344 patients with newly diagnosed AML enrolled into SWOG studies between 1986 and 2009, excluding Acute Promyelocytic Leukemia, illustrated this point dramatically. In that study, overall survival at 4 years was only 49% for patients with favorable cytogenetics; overall survival was approximately 25% for those with intermediate-prognosis cytogenetics, and only 9% for those with unfavorable cytogenetics [1]. Among the most important advances made in recent decades in the treatment of AML was the recognition of the exquisite sensitivity of Acute Promyelocytic Leukemia (APL) to differentiation therapy using all-trans-retinoic acid (ATRA). This observation was, shortly thereafter, accompanied by the identification of the Retinoic Acid Receptor Alpha (RARA) gene as a partner in the balanced translocation that drives APL. The dual recognition of a novel mechanism of disease—in the case of APL, overexpression of RARA, a protein that participates in proliferation and differentiation of hematopoietic cells—along with the identification of a compound, ATRA, that is a derivative of a vitamin, has provided a powerful paradigm for cancer therapy. This parallels the dramatic progress that has been made in the past fifteen years in the use of rationally designed, small molecules that target specific intracellular signal transduction pathways in cancer, best exemplified by the development of Imatinib, and followed by many new agents effective in a variety of solid tumors. The remarkable improvement in the rates of remission and survival for patients with APL has stimulated our laboratory, and many others to explore a similar approach for the treatment of non-APL subsets of AML, using vitamin D and analogs (collectively referred to henceforth as vitamin D and Deltanoids—“VDD”). Investigations have focused on either VDD alone, or in combination with other compounds, in an effort to induce differentiation of leukemia cells, and allow for less toxic chemotherapy, as is the case now in the treatment of patients with APL. The observation that vitamin D can induce acute myeloid leukemia blast cells *in vitro* to differentiate along the monocytic lineage was made approximately 30 years ago [2]; however, it remains to translate this observation into a clinically meaningful strategy. The barriers to translating this observation from the bench to the bedside include the induction of hypercalcemia VDD, as well as the need to identify which, if any, subset of patients with AML will be sensitive to VDD. The current understanding of the biologic basis for VDD effects in AML *in vitro* and *ex vivo* has recently been reviewed in detail [3]. The following is a review of published clinical experience that has accumulated to date regarding the use of VDD either alone or in combination with cytotoxic agents, other differentiating agents, or both. In many of these reports, the investigators included patients with a diagnosis of myelodysplasia (MDS), as well as AML, in their treatment cohorts, and for this reason, the effects of VDD in MDS patients are included in the discussion below.

## 2. Clinical Reports of Single-Agent VDD Treatment

Given the *a priori *expectation that there would be a low probability of a favorable clinical response to the use of single agent vitamin D or its derivatives to treat AML, it is readily understandable that there are few published reports of their use as single agents in such patients. However, in the late 1980's investigators at Kagawa Medical School in Japan reported a series of 3 patients treated using oral administration of from 4.5 up to 15 micrograms/day of 1 alpha (OH) vitamin D3 [4]. Two of these patients carried a diagnosis of AML, and the third patient had a diagnosis of the myelodysplastic syndrome refractory anemia with excess blasts. The authors reported a decrease in bone marrow blast percentage for each of the three patients, with transient hypercalcemia developing in one patient and resolving within three days of cessation of vitamin D3. Shortly thereafter, Takahashi and colleagues [5] reported their experience using alfacalcidol in a cohort of 13 patients, of whom two patients had AML—one patient with APL, and one with FAB-M4 histology by report. Patients received single-agent alfacalcidol at doses that ranged from 0.25 microgram/day up to 10 microgram/day orally, for at least one month of therapy; drug was administered only intermittently in order to avoid hypercalcemia. The authors reported that the patient with AML FAB-M4 had a minor response, utilizing response criteria developed by Koeffler [6]; the patient with APL did not respond. Of note, among the patients with a diagnosis of MDS, there were 3 partial responses and 2 minor responses. Nakayama reported a single case of AML responding favorably to treatment using 1 alpha(OH)D3 in a brief communication [7]. In this report, the patient was an 81-year-old man with pancytopenia on presentation, whose initial marrow exam reportedly showed 20% myeloblasts. He was treated using 1 alpha (OH) vitamin D3 at a dose of 6 micrograms daily by mouth as single-agent therapy. He achieved a normalization of blood counts by 4 weeks of therapy, with normalization of marrow morphology. The response was lost after the dose of 1 alpha (OH) vitamin D3 was tapered, and reescalation of the dose failed to achieve a second response. Hypercalcemia did not occur. These appear to be the only reports published to date of single agent VDD used as therapy for AML. 

There are at least six reports in the literature of single agent VDD treatment of patients with a diagnosis of myelodysplastic syndrome. In 1985, Koeffler, a leading investigator in this field, and colleagues administered 2 microgram/day of 1,25(OH)2D3 to 18 patients with myelodysplastic syndrome in an attempt to improve their hematopoiesis [6]. They reported that during therapy, peak peripheral blood granulocyte, platelet, and macrophage concentrations were slightly elevated as compared to baseline levels. Eight patients had a partial or minor peripheral blood response during the administration of 1,25(OH)2D3. However, no patient showed significant improvement of peripheral blood cell or marrow blast cell counts by the end of the study (greater than or equal to 12 weeks) as compared to their starting levels. Nine of the 18 patients developed hypercalcemia. In this paper, *ex vivo* treatment of dysplastic myeloid cells did demonstrate differentiation in response to 1,25(OH)2D3. In the 1989 report of Takahashi and colleagues from Japan discussed earlier, in addition to treating two patients with AML using alfacalcidol at doses ranging from 0.25 microgram/day up to 10 microgram/day orally for at least one month, there were 11 patients with various subtypes of myelodysplastic syndromes in the FAB classification schema. Of these, three patients showed partial responses, 2 patients showed minor responses and remainder of the patients did not respond. The hematological improvement of 6 responders was transient, reported by the authors to range from 1 to 2 months; however, one patient with low-risk MDS demonstrated an improvement in blood counts that persisted for more than 1 year ([5], op cit). In 1991, Motomura and colleagues published a clinical trial in a series of 30 patients with myelodysplasia. In 15 patients, 4–6 micrograms/day of 1-hydroxyvitamin D3 was administered, for a median duration of 17 months; a control group had no therapy. Leukemia-free survival of the D3-treated group was reported to be statistically superior to the control group; 7 patients in the control group developed acute leukemia, in contrast to only one in the D3 treated group [8]. In 1998, Mellibovsky and colleagues [9] described their experience in treating a series of 19 patients with low-to-intermediate grade MDS, as determined by the Bournemouth criteria [10], using single agent VDD. The first five patients in this study received 266 microgram calcifediol three times per week; the remaining twelve patients received Calcitriol, in doses ranging from 0.25 microgram/day increasing up to a maximum of 0.75 microgram/day by mouth, provided that plasma calcium concentrations remained in the normal range. Of the total of nineteen patients, 12 were men and seven women; mean patients age was 74 years. Patients were categorized by the FAB classification scheme; seven had refractory anaemia with ringed sideroblasts, five had refractory anaemia, one had refractory anaemia with excess of blasts, and six had chronic myelomonocytic leukemia. All the patients were in a low to intermediate risk group. Mean follow-up period was 26 months, range 9–75. Responders were defined as having experienced either a granulocyte or platelet count increase by 50%, hemoglobin increase of 1.5 g/dL, or transfusion needs decreased by 50%. Response was observed in 11 of the 19 patients. In the calcifediol treated group, one case responded, three were nonresponders, and one showed progression of disease. One responder was maintained on calcifediol for two years, stopped at his own request, and experienced a fall in hemoglobin, which then improved after resuming calcifediol. In the calcitriol group, 10 were responders (two with major response), and four were nonresponders. No correlation was observed between baseline levels of vitamin D metabolites and the presence of response. No hypercalcaemia was observed. These authors concluded that treatment with VDD could induce long-standing responses in blood counts in some low-intermediate risk MDS patients without inducing hypercalcemia.

In 1993, Yoshida and colleagues published a multicenter randomized clinical trial of alfacalcidol (1 alpha hydroxyvitamin D3) meant to evaluate the therapeutic effect of alfacalcidol in patients with MDS. Twenty-three evaluable patients were randomized to receive either a single daily oral dose of 6 micrograms of alfacalcidol, or supportive care as a control. Treatment was continued, whenever possible, for a period of 6 months. Response was assessed by weekly blood counts, clinical course, and repeated marrow examinations. No significant difference was noted between the alfacalcidol and control groups [11]. Three of the 13 patients in the alfacalcidol group and two of the 10 patients in the control group experienced progression of disease. One patient with refractory anemia showed a favorable response to alfacalcidol in all three hematopoietic cell lineages; the response, however, was not maintained, and discontinuation of the drug resulted in a worsening of pancytopenia which was refractory to a second course of alfacalcidol therapy. Hypercalcemia was the major toxic side effect of alfacalcidol therapy. These authors concluded that single-agent alfacalcidol therapy does not yield a significant clinical beneficial in patients with MDS. Most recently, Juckett and colleagues at the University of Wisconsin published a phase II clinical trial of single agent doxercalciferol, a vitamin D2 analogue, in 15 patients with MDS [12]. Patients were treated using doxercalciferol 12.5 microgram by mouth daily for 12 weeks. Nine of 15 patients completed the prescribed course, and of these six had stable disease. No patient had a favorable clinical response based upon International Working Group criteria, and eight patients experienced progressive disease while on therapy. Of interest, however, two patients with chronic myelomonocytic leukemia (CMML) had a marked rise in monocytes documented while on study, and one patient experienced hypercalcemia. These authors concluded that a twelve-week course of therapy using single-agent doxercalciferol has very limited activity in patients with MDS.

## 3. Clinical Reports of VDD Combined with Other Agents

Several groups have reported the clinical use of VDD in combination with other agents intended to force differentiation of pathologic myeloid progenitor cells, in a few series without the inclusion of classical cytotoxic antineoplastic agents. Most commonly, a VDD has been combined with a retinoid, with or without, a third agent or even a fourth agent. In 1991, Blazsek and colleagues in France reported their experience in treating two patients; one patient had APL in relapse and responded to single-agent all-trans-retinoic acid (a relatively new observation as of that writing). However, the second patient that they described in that report carried a diagnosis of MDS, and experienced a sustained hematologic response to treatment using the combination of prednisone with 1 alpha,25-dihydroxyvitamin D3 (1 alpha,25D3) as well as 13-*cis*-retinoic acid [13]. In this paper, 1 alpha,25D3 was administered at a dose of 0.25 micrograms by mouth three times per day for 30 days, in combination with prednisone 40 mg per day for the first 15 days and *cis*-Retinoic Acid 20 mg by mouth daily for 30 days. This patient continued the vitamin D for an 8-month period and the retinoic acid for a total of two and one-half years, apparently with a sustained response. 

In 2007, the Finnish Leukemia Group reported the results of a phase II clinical trial of the combination of valproic acid together with 13-*cis*-retinoic acid and 1,25-dihydroxyvitamin D3 in the treatment of MDS, including several cases chronic myelomonocytic leukemia and several cases of refractory anemia with excess blasts-2 (RAEB-2). Valproic acid, an antiseizure medication, has pleotropic effects, including inhibition of the P-glycoprotein; however, it was chosen in this instance because of the observation that Valproic Acid can inhibit histone deacetylase activity. Oral Valproic acid was titrated to achieve serum concentrations between 500 and 700 micromolar, and 13-*cis*-retinoic acid was administered at a dose of 10 mg by mouth twice daily. The VDD used in this study was Etalpha at a dose of 13 microgram by mouth daily. There were no episodes of hypercalcemia observed, although the majority of patients experienced the hypertriglyceridemia associated with the use of 13-*cis*-retinoic acid. Three of the nineteen patients experienced some hematologic improvement—a rise in hemoglobin and neutrophil count in one patient with chronic myelomonocytic leukemia, an improvement in platelet count in one patient with RAEB-2, and an improvement in hemoglobin level in one patient with refractory cytopenias with multilineage dysplasia [14]. 

A number of groups have attempted to combine vitamin D or its analogs with low-dose conventional antineoplastic chemotherapy in an effort to improve blood counts in patients with either AML, MDS, or both. Most commonly, Cytarabine has been used in combination with a VDD, sometimes with additional agents added to the regimen. In 1988, Hellström and colleagues in Sweden reported a series of 62 evaluable patients with either MDS or AML who were treated with various combinations of lowdose Cytarabine, alpha-interferon (IFN), 1 alpha-hydroxyvitamin D3 (vit D3), and retinoic acid. The Cytarabine was dosed initially at 15 mg/meter squared body surface area subcutaneously daily, and the interferon was administered initially at 3 million units subcutaneously daily. The 1 alpha-hydroxyvitamin D3 was dosed at 1 microgram per day by mouth in two divided doses, escalated until mild hypercalcemia was noted, with the dose then adjusted to maintain a serum calcium below 2.90 millimoles/liter. The overall response rate was 44%. Of these, 50% responded favorably to the combination of IFN, vitamin D3, and retinoic acid, a combination that they termed IDR. This was felt by the authors to be comparable to the response rate of 43% for low-dose Cytarabine alone [15]. 

The same group then performed a prospective, randomized Phase III clinical trial, in which 63 evaluable patients with myelodysplastic syndromes and 15 with acute myeloid leukemia were randomized between low-dose Cytarabine (arm A) as a single agent, and low-dose Cytarabine in combination with 13-*cis*-retinoic acid (13-CRA) and 1 alpha-hydroxy-vitamin D3 (1 alpha D3) (arm B). The doses of these agents were the same as used in their prior report described above ([15], op cit). The authors stated that 69 patients were evaluable and of these 18 (26.1%) responded to therapy [16]. The addition of 13-CRA and 1 alpha D3 had no significant favorable influence on neither survival of the patients, remission rates, nor duration of remissions. 12/27 patients (44%) in arm A and 6/29 patients (20%) in arm B progressed from MDS to AML during the course of the study (*P* = 0.0527). Arm B gave significantly more sideeffects than arm A (*P* = 0.005). The authors concluded that a clear cut therapeutic effect of the addition of 13-CRA and 1 alpha D3 to Cytarabine on MDS was not supported by this study. However, they also stated their interpretation that an inhibitory effect on AML development—that is, progression from MDS to AML—in some MDS subgroups could not be excluded by their data [16]. 

De Rosa and colleagues in Italy reported, in 1992, their experience treating forty-four patients with high-risk primary myelodysplastic syndromes. This cohort was treated with a combination of low-dose Cytarabine, retinoic acid, and vitamin D3 [17]. Morphological subtypes, using the FAB classification schema, were refractory anemia with excess of blasts (RAEB) in 16, RAEB in transformation (RAEB-T) in 20, and chronic myelomonocytic leukemia (CMML) in eight patients. In this series, Cytarabine was administered at a dose of 10 mg per meter squared body surface area twice daily for 15 consecutive days. The 13-*cis*-retinoic acid was given at a dose of 20 mg per meter squared body surface area for 21 days by mouth, and vitamin D3 was given at 0.75 microgram per day in three divided doses of 0.25 microgram per oral dose, also for 21 days. Cycles were repeated every four to six weeks. The therapy was continued in responders until relapse or death. The results were compared to those of a matched control group of 44 patients given supportive care only. In the treated group the overall response rate was 50% (75% in RAEB, 50% in RAEB-T, and 0% in CMML) and the survival was significantly better in the control group (*P* < 0.025). Comparing separately each FAB subgroup suggested that the treatment prolonged the survival in the RAEB-T subgroup (*P* < 0.002), but not the other two groups. The median duration of response was 15 months and the survival in responders was statistically better than in nonresponders (*P* < 0.0001). Myelosuppression was the most important side effect; however, no deaths related to treatment were noted. These authors concluded that this approach was useful for treatment of patients with high-risk myelodysplasia. 

Several years later, Ferrero and colleagues from Italy described their results in treating 53 MDS patients with a combination of *cis*-retinoic acid (cRA, 20 to 40 mg/day) and 1,25 alpha (OH)2 cholecalciferol [(OH)2D3, 1–1.5 micrograms/day] with or without intermittent 6-thioguanine (30 mg/m^2^/day). The 6-thioguanine was administered only to patients with bone marrow blast counts greater than or equal to 5%. The authors reported that treatment was well tolerated, without major toxicity. Among 25 patients with bone marrow blasts less than 5%, they observed one complete response, eight partial responses, and four minor responses (overall response rate 52%) with a median response duration of 8 months (2 ± 24). Median survival, which did not correlate with response, was projected by the authors, as of the time of publication, to be 76 months overall [18]. Thirty-one patients with BM blast excess (> or = 5%), including three of the previous group who progressed to refractory anemia with excess of blasts (RAEB), were treated with the three-drug protocol. One complete, 12-partial, and six minor responses were obtained (response rate 61%) with a median response duration of 6 months (2–29+). A significant difference in survival (*P* < 0.005) was observed between the 19 responders (median 25 months) and the 12 nonresponders (median 9 months). A reduction in the transfusion need was observed in 41% of the transfusion-dependent patients with blast excess and in 53% of those without blast excess. Therefore, these authors did conclude that combined differentiating therapy seems more effective than single-agent treatments. 

The same group subsequently reported their experience treating 26 patients with a diagnosis of AML, as well as 4 patients with MDS ineligible for standard chemotherapy. The regimen used was a combination of 13-*cis*-retinoic acid at 20 to 40 mg by mouth daily, together with dihydroxy-vitamin D3 (Rocaltrol) 1 microgram by mouth daily, along with 6-thioguanine 40 mg by mouth daily, and Cytarabine 8 mg per meter squared body surface area twice weekly by subcutaneous injection. The Cytarabine was administered during the initial two to three weeks of treatment. The median age of this group of patients was 72.5 years, and they had been deemed ineligible for standard chemotherapy. The response rate was 50%, with 27% complete remission. The median survival of the whole group and responders was 7.5 (1−47+) and 16.5 months (3.5−47+), respectively [19]. 

In a somewhat similar approach, Slapek and colleagues at Tufts University Medical Center published their experience in treating a series of twenty-nine patients, ranging from 62 to 82 years of age, all with a diagnosis of AML, treated using a 21-day course of continuous infusion Cytarabine at 20 mg per meter squared body surface area per day, together with 1,25-dihydroxyvitamin D3 (calcitriol) at a dose of 0.25 microgram by mouth twice daily. The calcitriol was continued until progression of disease or until the patient went off study. Hydrea was also given daily by mouth at a dose of 500 mg, started one day prior to the initiation of the Cytarabine infusion, and continued for 21 days as well. Ten patients had an antecedent myelodysplastic syndrome. Calcitriol was continued as the only postremission therapy. Thirteen patients (45%) obtained a complete remission, and 10 patients (34%) had a partial response for an overall 79% response rate [20]. There were three early deaths. The median remission duration was 9.8 months. Overall median survival was 12 months for all patients and 14 months for responding patients. All responding patients had marked bone marrow hypoplasia. Twenty patients received part or all of their chemotherapy as outpatients. These authors concluded that this regimen had acceptable toxicity and can result in prolonged remissions in elderly, high-risk patients with AML. 

Building upon work described above, Ferrero and colleagues in Europe recently reported on the use of recombinant human erythropoietin in combination with the previously investigated regimen of 13-*cis*-retinoic acid and dihydroxy-vitamin D3 in the management of anemia in MDS patients. They treated 63 MDS patients (excluding refractory anaemia with excess blasts, type 2 (RAEB2)) with the combination of 13-*cis*-retinoic acid and dihydroxy-vitamin D3 with, or without, the further addition of 6-thioguanine. Most patients were categorized as refractory cytopenia with multilineage dysplasia and RAEB1, in the WHO classification scheme, with intermediate 1 International Prognostic Scoring System (IPSS) score [21]. All patients had, at baseline, a hemoglobin of <9.5 gm/dL, and 70% required regular erythrocyte transfusions prior to beginning this therapy. The treatment dosing schedule included 13-*cis*-retinoic acid at 20 mg/day by mouth, and 1,25 di(OH) vitamin D3 at 1 microgram/day by mouth. Eleven of the 16 RAEB1 patients also received intermittent, low-dose 6-thioguanine (40 mg/d for 21 days every 5 weeks). rHuEPO (alpha epoetin in the majority, beta epoetin in a minority of patients) was added at different dosages and schedules according to practices of the different participating institutions and different time periods of treatment. Until 2002, 10,000 unit formulations only were available, and patients received weekly doses ranging from 10,000 units subcutaneously three times weekly up to 10,000 units subcutaneously daily. From 2002 onwards, 40,000 unit formulations of alpha erythropoietin became available, and dosages ranged from 40,000 units/week to 40,000 U every 3 to 4 days. Median weekly dose overall was 60,000 units per week (range: 30,000–80,000 units/week). All patients were treated for at least 6 months, and in the case of response, until disease progression or death. The treatment started within 12 months from diagnosis in 52 patients and between 15 and 48 months (median: 21) from diagnosis in 11 patients was well tolerated, and erythroid response rate according to new International Working Group criteria [23] was 60%:50% in RAEB1 and 64% in non-RAEB patients. The weekly recombinant human erythropoietin dose administered ranged from 30,000 units per week to as much as 80,000 units per week. Median response duration was 16 months, and median survival reached 14 months for RAEB1 and 55 months for non-RAEB patients, with a significant difference in the latter between responders and nonresponders (median 82 months versus 44 months; *P* = 0.036). No patient experienced clinically significant hypercalcemia. 

In a recent novel approach, Akiyama and colleagues in Japan recently reported the results of a Phase II trial of the combination of either vitamin K monotherapy, or vitamin K in combination with 1-alpha hydroxy-vitamin D3 for the treatment of patients with either low or intermediate-1 risks MDS. A total of 24 patients were enrolled into this study in total [22]. The overall response rate to vitamin K monotherapy (45 mg/day) after 16 weeks was 13% (5/38) including 4 cases with improvement of both anemia and thrombocytopenia and improvement in 1 case with thrombocytopenia. They then enrolled and evaluated 20 out of 33 vitamin K-monotherapy nonresponders for vitamin K plus vitamin D3 (0.75 microg/day) combination therapy. The overall response rate at 16 weeks after initiation of vitamin K plus vitamin D3 was 30% (6/20). Hematologic improvement for hemoglobin (Hb) was observed in 6 out of 11 patients (55%) and Hematologic improvement for thrombocytopenia in 3 out of 11 patients (27%), respectively. No Hematologic Improvement was observed for neutropenia in vitamin K monotherapy, nor in response to vitamin K plus vitamin D3 combination therapy. There was a suggestion in the data that International Prognostic Scoring System for MDS scores and absolute neutrophil counts positively correlated with favorable response, and Hemoglobin levels inversely correlated with the response to vitamin K plus vitamin D3 combination therapy. These authors concluded that vitamin K plus vitamin D3 combination therapy appears to be a promising treatment approach for improving anemia and thrombocytopenia in MDS patients with low/intermediate-1 MDS.

## 4. Discussion

A summation of the numbers of patients treated in the reports cited above documents that more than 85 patients with a diagnosis of AML, and a total of more than 350 patients with a diagnosis of MDS, have been treated using a vitamin D derivative either alone, or in combination with additional agents—as is evident from Table 1. Among this population, response rates have been highly variable, and this may be attributed to several factors. AML is a heterogeneous disease, with dramatic differences in the biologic subtypes, both with respect to the mutational events that drive the disease, as well as the prognosis of the disease based upon cytogenetics, molecular features, and the treatment paradigm employed. At one end of the spectrum, AML with core binding factor mutations generally have a very favorable prognosis when treated using high-dose Cytarabine as a part of therapy; at the other end of the spectrum, AML with MLL-1 gene mutations generally have an abysmal prognosis in the absence of allogeneic hematopoietic transplantation. Many of the reports reviewed herein were published prior to the recognition in the late 1990's of the core-binding factor mutations and their prognostic and treatment implications; this is similarly so for the impact of the MLL-1 gene, to identify just two of many important observations regarding AML. The heterogeneity of MDS is even greater with respect to biologic features, disease natural history, and prognosis, as compared to AML. How the biologic heterogeneity of these diseases impacts upon the clinical responsiveness of an individual patient has not been investigated to date, but it is very likely that adverse molecular events play a role in the relative unresponsiveness of some patients disease to vitamin D therapy. It is clear that some patients described in the clinical treatment reports above have benefited from vitamin D, or vitamin D analog therapy; however, dissecting the specific features that could predict responsiveness is difficult based on the relatively limited biologic data provided in the reports above. *A priori*, one might expect that patients with lower-grade (low IPSS score) MDS would be more likely to benefit from vitamin D therapy than patients with higher risk MDS; however, in the report of De Rosa and colleagues ([17], op cit), that was not the case, and patients with RAEB-T appeared more likely to benefit from vitamin D therapy, when compared to historical controls. However, this has not been formally tested to date in a prospective, randomized clinical trial with fully matched controls. The only prospective, randomized clinical trial summarized above was a small study, not powered to detect a modest impact of vitamin D, and randomized patients between low-dose Cytarabine (arm A) as a single agent, and low-dose Cytarabine in combination with 13-cis-retinoic acid (13-CRA) and 1 alpha-hydroxy-vitamin D3 ([16], op cit). Consequently, the effects, if any, of the vitamin D agent would have been obscured by the use of 13-CRA even if the numbers of patients had been larger.

A further issue in determining the efficacy of a VDD in the treatment of AML and MDS is the heterogeneity in the vitamin D agent chosen, which has varied from one report to another, as well as the dose and dosing schedule variations. The heterogeneity in the choice of vitamin D agent and the dosing schedule makes aggregating the data from the disparate trials extremely difficult. In order to glean more meaningful insight into the potential role for VDD in the treatment of AML and MDS, an optimal VDD, as well as an optimal dosing regimen, will ideally need to be defined in formal Phase I and Phase II studies, and then subjected to testing in a large, randomized Phase III clinical trial. *In vitro*, concentrations of vitamin D that induce differentiation are typically in the range of 1 nanomolar 1,25(OH)2 vitamin D3 [24]; however, conventional VDD administered in a dose that would achieve such a blood level carries a significant risk of inducing clinically significant hypercalcemia. Noncalcemic VDD agents have been developed [25], but to date have not been tested in patients with MDS or AML.

## 5. Summary

There is extensive preclinical data establishing the ability of vitamin D and its analogs to induce immature myeloid hematopoietic cells to terminally differentiate into mature monocytic cells, at which point these cells are no longer able to proliferate. However, in the 19 reports summarized in this paper, only a minority of patients experienced either a transient or a persistent improvement in blood counts in response to VDD-based therapy. The observation that a meaningful number of patients did experience some degree of objective, favorable response demonstrates the potential for clinical use of VDD as differentiation therapy in the management of MDS and AML. However, substantial progress remains to be made before this approach can be included in the formal armamentarium of treatments for MDS and AML.

## Figures and Tables

**Table 1:** Clinical reports of vitamin D in the treatment of patients with AML and MDS.

Vitamin D derivative	Dose schedule	Duration of therapy	Concurrent agents	Disorder treated	Number treated	Response	First author	Reference
1 alpha(OH) vitamin D3	4.5 to 15 microgram/day	4 weeks	Single agent	AML	2	Transient decline in marrow blasts	Irino	[4]
				MDS	1	Transient decline in marrow blasts		
Alfacalcidol	0.25 to 10 microgram/day	≥4 weeks	Single agent	AML	2	1 minor response	Takahashi	[5]
				MDS	11	3 partial and 1 minor response		
1 alpha(OH) vitamin D3	1 microgram/day	>4 weeks	Single agent	AML	1	1 major response	Nakayama	[7]
1,25(OH)_2_ vitamin D3	2 microgram/day	12 weeks	Single agent	MDS	18	8 transient, partial responses	Koeffler	[6]
1 hydroxy vitamin D3	4 to 6 microgram/day	17 months	Single agent	MDS	15	Improved PFS compared to control	Motomura	[8]
Calcifediol	266 microgram 3 days/week	Up to 2 years	Single agent	MDS	5	1 major response	Mellibovsky	[9]
Calcitriol	0.25 to 0.75 microgram/day	Up to 2 years	Single agent	MDS	14	10 responders, 2 major	Mellibovsky	[9]
Alfacalcidiol	6 microgram/day	6 months	Single agent	MDS	13	1 transient response	Yoshida	[11]
Doxercalciferol	12.5 microgram/day	12 weeks	Single agent	MDS	15	No formal response	Petrich	[12]
1,25(OH)_2_ vitamin D3	0.75 microgram/day	12 weeks	Prednisone + 13 cis-Retinoic Acid	MDS	1	1 major response	Blazsek	[13]
1,25(OH)_2_ vitamin D3	13 microgram/day	16 weeks	Valproic acid	MDS	19	3 major response	Siitonen	[14]
1 alpha(OH) vitamin D3	1 microgram/day	Variable	Cytarabine ± IFN ± Retinoids	AML	15	5 responses (including stable disease)	Hellström	[15]
1 alpha(OH) vitamin D3	1 microgram/day	Variable	Cytarabine ± IFN ± Retinoids	MDS	47	22 responses (including stable disease)	Hellström	[15]
1 alpha(OH) vitamin D3	1 microgram/day	Variable	Cytarabine ± 13 cis-Retinoic Acid	MDS	69	13 responses	Hellström	[16]
1 alpha(OH) vitamin D3	1 microgram/day	Variable	Cytarabine ± 13 cis-Retinoic Acid	AML	15	5 responses	Hellström	[16]
1,25(OH)_2_ vitamin D3	0.75 microgram/day	Variable	Cytarabine + 13 cis-Retinoic Acid	MDS	44	22 responses	De Rosa	[17]
1,25(OH)_2_ vitamin D3	1 to 1.5 microgram/day	Until progression	13 cis-Retinoic Acid + 6TG	MDS	31	19 responses (patients with >5% blasts)	Ferrero	[18]
Dihydroxy-vitamin D3	1 microgram/day	Until progression	Cytarabine + 13 cis-Retinoic Acid	AML	26	15 responses among all treated	Ferrero	[19]
Dihydroxy-vitamin D3	1 microgram/day	Until progression	Cytarabine + 13 cis-Retinoic Acid	MDS	4	15 responses among all treated	Ferrero	[19]
1,25(OH)_2_ vitamin D3	0.5 microgram/day	Until progression	Cytarabine	AML	29	23	Slapak	[20]
1,25 di(OH) vitamin D3	1 microgram/day	>6 months	13-cis retinoic acid, 6TG, Epo	MDS	63	38	Ferrero	[21]
1-alpha hydroxy-vitamin D3	0.75 microgram/day	16 weeks	Vitamin K	MDS	20	6	Akiyama	[22]
